# Transforming sepsis management: AI-driven innovations in early detection and tailored therapies

**DOI:** 10.1186/s13054-025-05588-0

**Published:** 2025-08-19

**Authors:** Praveen Papareddy, Thamar Jessurun Lobo, Michal Holub, Hjalmar Bouma, Jan Maca, Nils Strodthoff, Heiko Herwald

**Affiliations:** 1 Department of Laboratory Medicine, Biomedical Center, Lund University, BMC C14 Lund, Sweden; 2 Department of Clinical Pharmacy & Pharmacology, University Medical Center Groningen, University of Groningen, Groningen, The Netherlands; 3 Department of Infectious Diseases, First Faculty of Medicine, Charles University and Military University Hospital Prague, Prague, Czech Republic; 4 Departments of Clinical Pharmacy & Pharmacology, Acute Care and Internal Medicine, University Medical Center Groningen, University of Groningen, Groningen, The Netherlands; 5 Department of Anesthesiology and Intensive Care Medicine, Faculty of Medicine, Institute of Physiology and Pathophysiology, University Hospital Ostrava, University of Ostrava, Ostrava, Czech Republic; 6 School VI - Medicine and Health Services, Carl von Ossietzky University of Oldenburg, Oldenburg, Germany

**Keywords:** Artificial intelligence, Sepsis management, Precision medicine, Early detection, Clinical decision support

## Abstract

Sepsis remains a leading cause of mortality worldwide, driven by its clinical complexity and delayed recognition. Artificial intelligence (AI) offers promising solutions to improve sepsis care through earlier detection, risk stratification, and personalized treatment strategies. Key applications include AI-driven early warning systems, subphenotyping based on clinical and biological data, and decision support tools that adapt to real-time patient information. The integration of diverse data types, such as structured clinical data, unstructured notes, waveform signals, and molecular biomarkers, enhances the precision and timeliness of interventions. However, challenges such as algorithmic bias, limited external validation, data quality issues, and ethical considerations continue to hinder clinical implementation. Future directions focus on real-time model adaptation, multi-omics integration, and the development of generalist medical AI capable of personalized recommendations. Successfully addressing these barriers is essential for AI to deliver on its potential to transform sepsis management and support the transition toward precision-driven critical care.

## Introduction

### Introduction

Sepsis is a dysregulated response to infection that can lead to life-threatening organ dysfunction [1], which affects nearly 50 million people worldwide annually [2]. Advances in critical care have increased in-hospital survival to 85% [3–5]. Sepsis survivors have excess long-term morbidity [5] and a 5-year survival rate of 60% [6]. Despite advancements in many other medical areas, sepsis remains a complex and elusive condition to recognize in its early stage and treat effectively. The host response to the infection involves complex interplays, such as the interaction between the immune response and the coagulation system. If systemic decompartmentization of inflammation from the original focus of infection occurs, patients may often suffer from arterial hypotension, distributive shock, and widespread coagulation impairment, such as disseminated intravascular coagulopathy, leading to multiple organ failure development [7, 8]. Conversely, immune system hyperresponsiveness may occur in some patients, leading to overwhelming infection. This immunological imbalance is now considered a hallmark of sepsis pathogenesis and a target for precision medicine approaches [9].

### Clinical challenges

Clinical studies have demonstrated higher survival rates of septic shock associated with earlier administration of antibiotics, this emphasizes the importance of rapid intervention to decrease mortality and morbidity [10]. Patient heterogeneity adds to this complexity, with individual responses to infection varying based on genetics and health status. For instance, patients with underlying diseases such as diabetes mellitus, immunodeficiency, autoimmune diseases, chronic lung, kidney, or liver diseases, and cancer, as well as individuals at the extremes of age including newborns and the elderly, are more susceptible to severe infections and the development of sepsis. Recent advances in sepsis research underscore the need for molecular stratification of these patients to tailor treatment strategies [11].

Antimicrobial resistance (AMR) is a growing concern globally that complicates sepsis treatment. In some countries, the emergence of resistant pathogens has severely limited treatment options, often leaving clinicians with few or no effective therapies. As a result, resistant infections significantly contribute to the mortality burden, accounting for an estimated 1.27 million deaths worldwide annually [12]. Early diagnosis and individualized treatment are essential for improving outcomes, yet current approaches often fail to address the heterogeneity of patient responses [13].

In 2021, *Critical Care Medicine* and *Intensive Care Medicine* published new guidelines for sepsis management, called The Surviving Sepsis Campaign [14], offering clinicians state-of-the-art instructions for treating patients with sepsis or septic shock. The guidelines stress the importance of early identification and appropriate management in the initial hours after sepsis development. As mentioned before, timely intervention in sepsis care is crucial, as for each hour delay of proper treatment, mortality increases by 7.6% from the onset of hypotension [15]. To this end, early administration of antimicrobials and adequate hemodynamic resuscitation using fluids and vasopressors depending on the patient’s needs, ideally within one hour of recognition, is essential for patients with hypotension and sepsis. The sepsis management guidelines recommend early elimination of the infection focus, including empiric administration of a combination of broad-spectrum antibiotics and, when necessary, surgical intervention such as incision and drainage of abscesses. It is important to note that samples for microbial analysis should be taken prior to antibiotic administration to increase the diagnostic yield [16]. Additional treatment strategies addressing vascular leakage and endothelial dysfunction are also under investigation to stabilize circulatory collapse in sepsis [17]. Sepsis-induced coagulopathy further complicates management and has been linked to poor outcomes in multiple studies [18].

### Role of AI in sepsis care

Advances in AI have opened new opportunities for precision medicine in sepsis care, offering tools to address the unique diagnostic and treatment challenges posed by this highly heterogeneous and time-sensitive condition. AI-driven models show promise in enhancing early detection, risk stratification, and treatment personalization [19]. By analyzing large datasets from clinical and molecular sources, AI holds potential for enabling more tailored interventions in sepsis. However, clinical implementation and evidence for improved outcomes remain limited at present [20].

This narrative review outlines three potential use cases for AI in sepsis care, followed by a discussion of key challenges that must be addressed to improve AI applications in this field. It then explores promising future directions before concluding with final remarks. The focus is on adult sepsis management, reflecting the current clinical definitions (including Sepsis-3) and the predominant application of AI technologies in adult patient populations.

While several reviews have explored the promise of AI in sepsis management, focus mainly either on early detection models [21, 22] or on specific algorithmic techniques [23, 24], but to a less extend aspects such as clinical translation, regulatory evaluation, and real-world implementation. This review aims to address that gap by providing a structured synthesis of AI applications across the sepsis care continuum, from early prediction and phenotyping to therapeutic guidance and deployment in clinical settings, while also explicitly addressing associated challenges and promising directions for future research.

## Use cases for AI in sepsis care

AI technologies show promise for enabling earlier interventions, more precise patient categorization, and personalized treatment strategies. However, these applications remain largely investigational and require further validation before widespread clinical adoption. Three critical use cases of AI in sepsis care include early detection, sepsis subphenotyping for risk stratification, and targeted treatment.

### AI in Early Detection and Early Warning Systems

One of the most promising applications of AI in sepsis management is its ability to detect the condition at an early stage. This may enable earlier identification of patients at risk and demonstrates strong predictive performance; however, evidence for improved clinical outcomes resulting from the use of such models remains limited [25].

Numerous AI-driven early warning algorithms for sepsis detection have been developed and evaluated in diverse clinical settings. One of the more extensively studied sepsis prediction tools is the InSight algorithm, which was initially developed and internally validated using retrospective data [26]. While that study demonstrated promising performance metrics, it did not compare InSight to traditional scoring systems. A subsequent prospective clinical trial showed that InSight implementation was associated with earlier sepsis detection and reduced ICU length of stay compared to usual care [27]. In addition to early detection of sepsis, AI-driven warning algorithms have been explored as tools to guide the timing of critical interventions, such as renal replacement therapy in patients with acute kidney injury related to sepsis. Retrospective analyses suggest potential associations between earlier AI-triggered intervention and improved outcomes, but prospective evidence remains limited and inconclusive [28]. The validation and implementation of these systems remain challenging. Diagnostic suspicion bias and a lack of rigorous validation can undermine the generalizability and real-world effectiveness of machine learning-based sepsis detection tools [29].

Other AI models, such as the Sepsis Early Risk Assessment (SERA) algorithm and the Targeted Real-Time Early Warning System (TREWS), process large datasets in real-time to enhance early sepsis detection. SERA combines structured data (e.g., vital signs) and unstructured clinical notes using Natural Language Processing (NLP) to predict sepsis up to 12 h before onset [30]. TREWS continuously monitors patient data, flagging potential sepsis cases before they become clinically apparent. A prospective evaluation suggested that early alert confirmation (within 3 h) was associated with faster treatment initiation, though the absence of a non-TREWS control group and potential confounders limit causal interpretation [31]. Subsequent analyses have raised additional concerns, including exclusion of false detections and cases where antibiotics were administered prior to the alert [32]. Both SERA and TREWS have demonstrated strong predictive accuracy, with reported AUC values of 0.94 and 0.97, respectively. The SERA algorithm’s performance is based on single-center internal validation [33]. TREWS has undergone prospective, multi-center evaluation, but its reported AUC value was derived from internal validation datasets [34].

Despite promising results, concerns regarding external validation and clinical implementation remain [35]. The clinical benefits of automated sepsis early warning systems extend beyond individual studies. A recent systematic review and meta-analysis demonstrated that these systems significantly reduced hospital mortality, ICU admissions, and length of stay [36]. These AI systems streamline clinical workflows by automating the detection process, allowing healthcare professionals to focus on decision-making and patient care.

AI models such as the previously mentioned TREWS and SERA, and the Epic Sepsis Model, differ markedly in their core features, validation methods, and clinical applicability, reflecting substantial heterogeneity in both performance and methodological rigor. As summarized in Table 1, these tools vary widely in input data requirements, reported AUC values, positive predictive performance, and degree of prospective evaluation. Although AUC is a frequently used performance metric, it does neither reflect clinical utility at decision-relevant thresholds nor provide insights into the calibration of the model [37]. Models such as TREWS report high AUCs but relatively low PPV, which may limit their actionability in real-world settings and contribute to alert fatigue. While TREWS demonstrated mortality benefit in a large multi-center prospective study [34], models like SERA [33] and the AI Clinician [38] remain confined to retrospective evaluation. Conversely, the Epic Sepsis Model showed markedly lower performance in independent validation (AUC ~ 0.63), despite wide adoption [39]. More recently, COMPOSER has shown promising prospective precision using language model-enhanced inputs [40]. These findings underscore that metrics like AUC alone may obscure critical limitations such as poor calibration or excessive alert volume.

**Table 1:** Comparative overview of selected AI-based sepsis prediction models

Model	Input Data	Validation	AUC	PPV/NPV	Deployment	Main limitations	Reference
TREWS	Structured EHR	Prospective multi-center (5 hospitals, ~ 590,000 patients)	0.97 (internal); 0.88 (prospective)	PPV ~ 27%	Clinical use (U.S.)	High false alarm rate; site-specific calibration	[34]
SERA	Structured + unstructured clinical notes	Retrospective (internal + external test set)	0.94	Sens/Spec ~ 0.87 each	Not deployed	No prospective evaluation	[33]
Epic Sepsis Model	Structured EHR	External validation (Michigan study)	0.63	Very low PPV; ~109 alerts per true case	Deployed in Epic hospitals	Poor generalizability; unclear thresholding	[39]
AI Clinician	Structured ICU data	Retrospective (MIMIC-III)	~ 0.85 (policy evaluation)	–	Not deployed	No prospective or external validation	[38]
COMPOSER-LLM	Structured EHR + LLM-based predictors	Prospective pilot	–	PPV ~ 32–36%, Sens 68–72%	Early pilot	Limited scale; LLM robustness unproven	[40]

The table summarizes key characteristics of widely cited AI tools for early sepsis prediction, including data inputs, validation type, performance metrics, deployment status, and primary limitations based on current peer-reviewed literature

To summarize, early warning systems represents the most commonly addressed use case for AI in sepsis care with models that show a seemingly promising performance, however, often lacking external validation and with still largely uncertain clinical impact.

### AI-driven Phenotyping

The capacity of AI to manage the complexities of sepsis extends beyond early detection, offering a powerful tool for subphenotyping, which categorizes patients into subtypes based on clinical and biological characteristics. These subphenotypes are associated with differing risks of clinical outcomes and may require tailored therapeutic approaches that could alter the disease trajectory. This shift from standardized treatments to personalized strategies utilizes unsupervised learning algorithms to process large patient datasets, identifying clinically relevant patterns and suggesting adaptive treatment approaches tailored to subphenotypes, which may inform future improvements in clinical management and outcomes [41]. In this context, “phenotype” refers to observable clinical characteristics, while “subphenotype” denotes data-driven groupings identified through unsupervised learning techniques. To maintain terminological clarity, the term “endotype” is not used and molecularly defined variants are referred to as “subtypes”.

A notable example of AI-driven phenotyping is the identification of four distinct sepsis phenotypes, α (alpha), β (beta), γ (gamma), and δ (delta), through machine learning algorithms [42]. Factors like inflammatory markers, organ dysfunction, and mortality risk differentiate these phenotypes. For instance, the δ phenotype, characterized by severe organ dysfunction and high inflammation, is associated with the highest mortality rate. In contrast, the α phenotype, with less severe organ impairment, has better outcomes. Recognizing these phenotypes early may enable clinicians in the future to tailor treatments such as fluids, vasopressors, and antibiotics based on the specific needs of each patient group.

In addition to unsupervised clustering approaches for identifying sepsis phenotypes, trajectory-based methods have also been used to define subphenotypes based on longitudinal patterns of vital signs. These approaches have identified distinct temperature trajectory-based sepsis subphenotypes associated with differing host inflammatory responses and clinical outcomes [43]. Causal inference frameworks offer a complementary strategy aimed at discovering subgroups that respond differently to specific treatments. Recent work using causal inference techniques has demonstrated the potential to identify distinct sepsis phenotypes that exhibit differential responses to interventions, such as fluid resuscitation strategies [44].

Despite their promise, most AI-driven phenotypes remain observational and have not yet been incorporated into prospective interventional trials or decision-support systems [42]. Translational barriers include limited real-time identifiability, unclear therapeutic implications, and the lack of validated protocols linking phenotype classification to bedside interventions [43, 44].

To summarize, the attempt to identify sepsis (sub)phenotypes is a promising use case for AI in sepsis care to address the inherent heterogeneity of sepsis. However, the clear differentiation of phenotypes their connection to different therapy response and/or differences in hard outcomes remains challenging.

### Personalized treatment and AI-supported clinical Decision-making

AI plays a critical role in personalizing treatment by predicting how individual patients will respond to therapies. By analyzing historical data, real-time patient information (vital signs, lab results, clinical history), and outcomes, AI models help clinicians tailor interventions to maximize efficacy while minimizing risks. For example, predictive models can guide antibiotic selection or vasopressor timing and dosage based on the patient’s unique physiological state [45].

Systems like the AI Clinician use reinforcement learning to adjust fluid and vasopressor recommendations based on real-time data dynamically, optimizing treatment strategies in rapidly changing critical care settings [46]. The AI Clinician, first described in a seminal study applying reinforcement learning to sepsis treatment strategies, demonstrated the potential of AI systems to recommend optimal fluid and vasopressor dosing based on retrospective ICU data [47]. A recent prospective validation study, the OVISS trial, extended this approach by evaluating reinforcement learning–based treatment recommendations for vasopressors and fluids in real-world ICU settings. The study demonstrated clinical feasibility and higher adherence to AI-suggested regimens, with trends toward improved patient outcomes compared to clinician-administered care [48]. Tree Augmented Bayesian Networks (TAN) and Dynamic Treatment Regimes (DTRs) similarly help clinicians manage sepsis risks by providing real-time updates to treatment plans and adapting to short-term changes in patient conditions [49, 50].

Incorporating sepsis subphenotypes (such as α, β, γ, or δ) alongside real-time data further enhances personalized care by categorizing patients based on their clinical and biological characteristics. This ensures more aggressive care for severe phenotypes and conservative management for milder cases. Subphenotype-based treatment plans continuously adapt to evolving patient conditions, improving outcomes through timely interventions.

Looking forward, AI tools will increasingly integrate omics data, such as genomics, epigenomics, transcriptomics, proteomics, lipidomics, and metabolomics, to provide even more personalized treatment recommendations tailored to each patient’s molecular profile. To summarize, AI-supported clinical decision support holds the promise to provide individualized treatment recommendations supported by data. This requires counterfactual reasoning skills, which are challenging to implement. It is therefore the least developed use case for AI in sepsis care.

## Challenges for AI in sepsis care

AI in sepsis care faces many challenges, which we broadly categorize as foundational, methodological and deployment-related and discuss in detail the following paragraphs.

### Foundational Issues

#### Sepsis definition

Machine learning models for sepsis recognition often face challenges due to the inherent complexity of the condition and the variability in its presentation. The absence of a single “*gold standard*” definition for sepsis complicates model comparisons, though the Sepsis-3 criteria, based on SOFA score changes and organ dysfunction, are increasingly accepted. This variability is further compounded by different clinical settings (e.g., ED, general ward, ICU) and the specific endpoints used, such as sepsis versus septic shock [51]. Large clinical care datasets are often used to train models, but they frequently lack the specific parameters needed to calculate the SOFA score, hindering accurate case labeling. The electronic SOFA (eSOFA), which relies on more commonly available parameters, may enable more reliable labeling of sepsis cases within such datasets [52]. Awareness of these seemingly subtle differences is essential, particularly when comparing models across different publications.

#### Evaluation challenges and external validation

A comparative evaluation of early detection models for sepsis represents a major challenge. Prediction scores are normally obtained from retrospective analyses, typically measured at the encounter level using data collected within a pre-specified window prior to sepsis onset [13, 14]. Dataset characteristics, including balancing, sepsis prevalence, and case definition criteria, strongly influence these performance metrics [33, 34]. The AUC is often used as a measure of overall predictive performance; however, clinical implementation requires additional metrics, such as sensitivity and positive predictive value (PPV), evaluated at alert thresholds relevant to clinical practice. These metrics are essential to assess the potential impact of false positives and false negatives on workflows and decision-making.

Although many AI models demonstrate impressive accuracy in controlled research environments, their real-world performance can be inconsistent. Differences in patient populations, healthcare practices, and data quality between hospitals limit generalizability​ [53, 54]. External validation of AI models across diverse settings is essential to build confidence in their reliability and ensure they can handle the complexity of real-world medical environments. Without rigorous validation, clinicians cannot rely on AI-driven tools to support clinical decision-making, further limiting their integration into clinical practice​ [55]. External and prospective validation across diverse settings is therefore critical to establish reliability before clinical integration. Several recently published multi-site studies have addressed this gap, and prospective implementation studies have reported promising outcomes [56–58], including reductions in mortality. Nevertheless, randomized controlled trials remain the gold standard and are still pending [59].

The risks of deploying models without rigorous validation are illustrated by the Epic Sepsis Model (ESM). Despite widespread adoption through Epic’s electronic health record, an external evaluation at the University of Michigan revealed that ESM missed 67% of sepsis cases, showing low sensitivity and raising serious concerns about its clinical utility. This case underscores the need for thorough external validation and prospective evaluation before integrating AI-driven prediction tools into clinical workflows [60].

#### Toward regulatory standards and prospective validation

As the clinical use of AI-based decision support systems in sepsis management expands, it raises a critical question: should these tools be held to the same standards of clinical validation as pharmacologic agents, diagnostic biomarkers, or medical devices? While most AI models are currently deployed after retrospective validation and occasionally external testing, few undergo the kind of prospective, real-world evaluation required for other clinical interventions.

Recent systematic evaluations of FDA-approved AI/ML-enabled medical devices show that fewer than 10% are supported by prospective clinical trials, and even fewer provide transparent reporting on model performance across patient subgroups or settings [61]. This stands in contrast to the standards expected for traditional interventions such as drugs or invasive monitoring devices, which require evidence from randomized controlled trials (RCTs) demonstrating clinical benefit, not just predictive accuracy.

Yet designing RCTs for AI interventions, especially those operating in the background or “silently”, raises unique challenges. In silent deployment, AI models may process data and generate risk predictions without alerting clinicians, allowing for unobtrusive evaluation of model performance. However, this setup complicates the application of clinical equipoise and informed consent, since patients may be randomized to arms where potentially actionable insights are withheld. These challenges raise profound ethical concerns, particularly around transparency, patient autonomy, and fairness, as silent deployment may undermine informed patient choice and institutional accountability. Moreover, silent trials blur the boundaries between observational and interventional research, complicating regulatory classification and challenging the oversight mechanisms typically used for clinical investigations. A robust ethical framework is needed to guide the design of such studies, including mechanisms to safeguard patient rights even when disclosure is limited. In light of these difficulties, alternative approaches rooted in causal inference have been proposed, in which emulated target trials using real-world data, such as EHRs, can approximate RCT conditions. For example, it has been reported that replicates RCT results using observational data, highlighting how carefully designed causal models can offer valid treatment effect estimates when traditional RCTs are infeasible or ethically constrained [62]. Such frameworks may be especially valuable in the context of AI systems deployed silently, where prospective experimentation is hindered by regulatory ambiguity and consent challenges.

From a methodological standpoint, these trials often rely on complex causal inference frameworks to compare outcomes across AI-assisted and standard care arms, yet such comparisons are sensitive to unmeasured confounding, patient heterogeneity, and context-specific clinical workflows [63]. However, these causal inference findings remain hypothesis-generating, are limited by unmeasured confounders and the static nature of current phenotyping approaches in this dynamic disease, and are not yet validated bedside strategies; prospective randomized trials are essential to confirm their clinical utility. Moreover, if clinician behavior is influenced, even unconsciously, by the presence of AI tools in the system, true isolation of effects becomes elusive. As such, while RCTs remain the gold standard for clinical validation, alternative designs, such as stepped-wedge trials or quasi-experimental models with robust sensitivity analyses, may offer pragmatic pathways for evaluating AI in dynamic and ethically sensitive settings.

A growing body of literature calls for more rigorous and standardized frameworks for evaluating AI in healthcare. These frameworks advocate for adaptive, risk-based regulatory approaches but emphasize the need for prospective evidence of clinical utility prior to deployment [64–66]. This perspective is especially relevant in sepsis care, where real-time decisions about fluids, antibiotics, and vasopressors can have immediate consequences for patient outcomes.

A limited but growing number of AI-based systems for sepsis have undergone prospective evaluation. For example, the NAVOY^®^ Sepsis algorithm was recently assessed in a randomized controlled trial using Sepsis-3 criteria and demonstrated a statistically significant improvement in early sepsis identification and timely treatment initiation [67]. A multimodal large language model–driven system was prospectively evaluated in ICU settings, demonstrating robust real-time performance and workflow integration [68]. Another example, the Sepsis Watch model, has undergone multisite external validation, illustrating both its potential and the challenges of reproducibility in diverse clinical environments [69].

Moving forward, AI systems intended for high-stakes decisions in critical care should undergo clinical validation standards similar to those applied to devices or diagnostics. This includes not only technical performance and fairness evaluations but also prospective trials to determine their actual impact on patient outcomes.

### Methodological limitations

#### Publication and reporting bias

The field of AI in sepsis care is subject to significant publication and reporting bias. Studies highlighting successful or high-performing models are more likely to be published, while those that fail to generalize or show limited clinical benefit often remain underreported. The failure of the widely deployed Epic Sepsis Model (ESM), which was only publicly scrutinized after an external evaluation revealed it missed 67% of sepsis cases, illustrates how such biases can distort perceptions of progress and delay critical scientific learning [70]. Learning from models that fail in external validation or real-world deployment is essential, as these cases often reveal hidden biases, fragile assumptions, or context-specific limitations that are crucial for developing more robust and equitable AI systems [71]. Failed models should therefore be systematically audited through post-hoc performance analyses, recalibration efforts, and transparent reporting practices to inform future model development and deployment strategies.

#### Transparency and explainability

The *“black box”* nature of many AI models, particularly deep learning systems, poses significant challenges for clinicians in trusting AI-generated recommendations. Various Explainable AI (XAI) techniques have been developed, often applied as post-hoc analyses to trained models. These methods identify the most influential input features and offer approximate insights into why a model made a specific prediction. However, they do not provide true transparency into the model’s internal logic or how it generalizes across different contexts.

Shapley-value-based methods are widely used for tabular data, and post-hoc attribution techniques have been adapted for physiologically relevant domains, such as time series data [24]. While these techniques highlight important input features, they offer only limited and sometimes incomplete insights into the model’s overall behavior. Thus, their direct benefit to end-users in clinical decision-making remains unproven, though XAI provides a promising foundation for model auditing and knowledge discovery [72]. To address current limitations, rigorous validation protocols similar to those used for drug approval have been proposed to evaluate model performance across diverse patient subgroups [73].

Explainable techniques that provide clear insights into AI recommendations can contribute to building trust and ensuring safe clinical use. However, achieving transparency and accountability will require broader approaches, including interpretability by design, clinical oversight, and governance frameworks [74–76]. This challenge is not unique to sepsis detection but also applies to AI-driven phenotyping, where classifications must be both actionable and understandable to clinicians. Opaque or overly complex phenotypes risk eroding trust and limiting adoption​ [77].

#### Algorithmic bias

AI models are only as good as the data on which they are trained, posing the risk of algorithmic bias. If training datasets do not adequately represent the diversity of patient populations, models may underperform in specific demographic groups, potentially leading to inequities in sepsis care where early detection is critical [55]. For example, an AI model trained primarily on data from one demographic might struggle to detect sepsis in patients from other ethnic or socioeconomic backgrounds, potentially leading to delayed or inappropriate treatment. AI systems must also mitigate biases in data, as these can lead to unfair treatment or discrimination [78]. Recent studies have shown that racial disparities in pulse oximetry accuracy, which lead to systematically underestimated hypoxemia in certain patient groups, can propagate through sepsis prediction models and amplify inequities in early detection [79].

Differences in the use of life-sustaining interventions such as mechanical ventilation, vasopressors, and renal replacement therapy across racial and ethnic groups, often associated with underlying socioeconomic disparities, have been documented in ICU patients with sepsis and may introduce confounding patterns into AI models trained on real-world critical care data [80]. Mitigation strategies, including subgroup-specific validation, bias-aware training, and continuous post-deployment auditing, are essential to ensure equitable sepsis care.

#### Data quality

Critical care data often contain missing values or inconsistent entries, which can significantly affect model predictions [81]. In high-stakes environments, even minor gaps can lead to unreliable AI-driven decisions. For example, predictive models in neonatal care have been shown to suffer from inconsistencies in patient records, skewing results and reducing reliability [82]. Incomplete or inaccurate data not only undermine AI-generated recommendations but also erode clinicians’ trust in these systems.

The predictive performance of AI models is directly linked to the completeness and accuracy of their training datasets. Limited data coverage and poor standardization remain major obstacles, particularly when integrating heterogeneous sources [83]. Improving data quality through standardized documentation, robust preprocessing, and advanced imputation techniques will be crucial to ensure reliable and clinically acceptable predictions.

### Deployment-related barriers

#### Real-world implementation

Integrating AI into existing healthcare systems poses both logistical and technical challenges. AI tools must be compatible with current EHR systems, which are often not optimized for advanced AI functionalities. AI algorithms not only demand sufficient computing power but also depend on the timely availability of input data in the appropriate format and resolution to function effectively. This requires substantial investment in infrastructure upgrades and coordination across departments [54]. Ensuring that AI systems enhance rather than complicate clinical workflows is key to fostering widespread adoption [54].

A further major barrier to real-world deployment is the clinical burden created by excessive alerts, which can strain healthcare resources and may even cause harm through unnecessary treatments. Even highly accurate models like TREWS can generate false alarms. This strains healthcare resources and may cause harm through unnecessary treatments​ [31]. False positives not only contribute to unnecessary interventions but can also lead to desensitization among healthcare providers, reducing their responsiveness to real alarms. This phenomenon, commonly known as alarm fatigue, has been widely documented in sepsis detection systems, where the balance between sensitivity and specificity must be carefully managed to minimize unnecessary alerts [84]. Efforts to improve sepsis detection models focus on reducing false positives by optimizing the timeliness of predictions and adjusting algorithms to weigh false alarms more heavily in the model’s training process. These improvements help ensure that alerts are triggered at the most clinically relevant times, thereby reducing alarm fatigue while maintaining early detection benefits [85]. In addition to these efforts, new models like COMPOSER are being developed to tackle alarm fatigue more effectively. The COMPOSER model leverages the formalism of conformal prediction, a technique that provides statistically robust out-of-distribution detection. This allows the model to identify samples that deviate significantly from the training data and refrain from making predictions in those cases, thus reducing the likelihood of false positives and improving overall prediction quality [86]. We would like to increase the readers’ awareness for not neglecting false positives compared to false negatives, which goes in hand with being transparent about the choice of the algorithm’s decision threshold. Approaches such as conformal prediction represent a promising innovative path to address this long-standing problem.

Finally, real-world implementation presents a significant hurdle. Models that perform well in controlled research environments may struggle in diverse healthcare settings. Healthcare institutions must invest in the necessary infrastructure and training to integrate these tools into daily practice [20]. Currently, no regulations exist regarding reimbursement for the use of AI models in clinical practice, despite the potential substantial costs incurred by manufacturers for their development, validation, and certification. Additionally, the economic burden of sepsis adds complexity, with hospital-related costs varying across healthcare systems [87]. Optimizing resource use and improving treatment efficiency through these advanced technologies could alleviate some of the financial strain.

In addition to economic and technical considerations, the successful deployment of AI systems must also address key principles from human factors engineering. Recent studies have highlighted how clinician perceptions of alert utility are shaped by cognitive load and prior experience, with less experienced providers often finding alerts more actionable and beneficial [88]. Moreover, effective implementation requires co-design with clinicians to reduce alert fatigue, support hypothesis generation, and ensure seamless workflow integration, features that have been shown to improve usability and clinical trust [89].

While the clinical promise of AI in sepsis care continues to grow, its real-world impact hinges on addressing critical economic and system-level barriers. Implementing AI tools requires more than technical validation, it also demands sustainable financing models, clear reimbursement pathways, and evidence of cost-effectiveness. Recent work has demonstrated the economic potential of AI-based sepsis prediction. For example, one study estimated that optimizing sepsis alert thresholds across diagnostic categories could result in nearly $4.6 billion in cost savings annually for the U.S. Medicare system, primarily by reducing unnecessary utilization and improving compliance at clinically relevant decision points [90]. Yet, cost-effectiveness analyses remain the exception rather than the norm. Few models undergo economic evaluation in parallel with validation studies, limiting stakeholders’ ability to weigh financial benefit against implementation cost and workforce burden.

Beyond quantifying potential savings, the adoption of AI systems in critical care is often limited by the lack of early health-economic analyses, limited evidence on cost-effectiveness, and uncertainty regarding real-world financial impact, factors that hinder informed investment and implementation decisions [91]. These systemic issues become particularly acute in under-resourced settings, where equity concerns compound the difficulty of deploying advanced AI infrastructure.

Another recent perspective argues that traditional evaluation metrics are insufficient for guiding AI deployment at scale. Instead, it proposes sociotechnical frameworks that integrate cost-benefit analysis with organizational readiness, data infrastructure maturity, and clinician acceptance [92]. From this viewpoint, demonstrating return on investment involves not only direct savings but also alignment with broader institutional goals, including quality improvement and staff efficiency.

Together, these findings suggest that the successful integration of AI in sepsis care will require a holistic approach that incorporates financial modeling, policy coordination, and system-level readiness, in addition to technical performance. Without attention to these economic dimensions, even well-validated tools may fail to deliver meaningful impact in clinical practice.

#### Data privacy

AI systems require access to vast amounts of data, often involving sensitive patient information sourced from electronic health records (EHRs), lab results, and physiological monitoring. However, concerns about data privacy and security are paramount, particularly in the context of compliance with regulations such as the General Data Protection Regulation (GDPR) and the Health Insurance Portability and Accountability Act (HIPAA). The challenge lies in balancing the utility of this data for AI models with the need to protect it from breaches or unauthorized access [93]. HIPAA mandates strict safeguards to protect personal health information (PHI) in both storage and transmission [94]. To address these concerns, advanced security measures such as encryption and access protocols are essential. Moreover, privacy-preserving techniques like federated learning, where models are trained on decentralized data without sharing raw information, offer a way to harness data for AI while maintaining strict privacy standards [95, 96]. Another promising approach is data synthesis, which involves generating artificial datasets that mimic the statistical properties of real patient data without exposing individual identities; this allows AI models to be trained and validated while mitigating privacy risks. Even when data is de-identified, re-identification risks persist, particularly when dealing with large and complex datasets [97]. Therefore, continuous vigilance and evolving strategies are necessary to ensure patient data remains secure. Transparency also plays a key role: patients must be informed about how their data is used and protected, fostering trust in AI-driven healthcare systems [95]. As AI technologies advance, the need for strong privacy frameworks becomes even more pressing. Balancing innovation with robust data protection will ensure that the promise of AI in healthcare does not come at the cost of patient privacy [94].

#### Ethical and legal considerations

Ethical and legal considerations, including compliance with GDPR and HIPAA, are essential for the safe implementation of AI in healthcare [93]. Beyond regulatory compliance, ethical challenges such as transparency, fairness, and clear accountability remain critical. The “black box” nature of many AI algorithms raises concerns about explainability, particularly when AI-driven decisions affect patient outcomes [93]. To foster trust, healthcare professionals and patients must understand how AI systems generate recommendations [98]. Equally important is defining responsibility and liability in the event of adverse outcomes, whether for developers, healthcare providers, or institutions [50]. The lack of clarity around accountability is a significant barrier to integrating AI into high-risk clinical settings like sepsis care [74, 99]. Regulatory frameworks also play a decisive role. In the European Union, the Medical Device Regulation (MDR) requires CE certification for AI systems classified as Software as a Medical Device (SaMD), ensuring compliance with strict safety, performance, and risk management standards. In the United States, the Food and Drug Administration (FDA) regulates AI-based medical devices through pathways such as De Novo and 510(k), which assess safety, efficacy, and equivalence to existing technologies. Although these requirements add complexity and cost, they are crucial to ensuring patient safety and public trust. A multi-faceted approach addressing ethical, legal, and regulatory dimensions is essential for the safe integration of AI into healthcare systems.

## Future directions for AI in sepsis management

AI is already transforming sepsis management, offering advancements in detection, diagnosis, and treatment. As AI tools and multimodal data integration evolve, they will enable more personalized, real-time interventions to address the complexities of sepsis care.

### Extending input modalities: From clinical routine data to the integration of unstructured text, OMICs data, and raw waveforms

#### Routine clinical data

Most sepsis prediction algorithms rely on routine clinical data such as patient demographics, vital signs, and laboratory values [100]. For instance, a recent meta-analysis has identified several critical factors that contribute positively to sepsis prediction scores [101]. These factors include heart rate, respiratory rate, temperature, and various laboratory and blood gas values, all closely in line with clinical expectations. Moreover, neural network-based predictors have emerged as the predominant model category for sepsis prediction [102]. However, direct model comparisons across different publications remain difficult due to the heterogeneity of clinical endpoints and clinical settings. This urges for the creation of benchmark datasets as indicators for measurable performance improvements in the field, along the lines of standardized benchmarks for performance evaluation [103].

To improve the predictive accuracy, the inclusion of additional input variables is essential, as recent studies have shown. These include unstructured text such as progress notes, nursing notes, chief complaints, diagnostic reports, or discharge summaries, which may play a potential role in sepsis prediction [104]. Most approaches rely on traditional features from natural language processing such as n-grams, Latent Dirichlet Allocation topic modeling, or, more recently, word vectors or pre-trained text encoders of the BERT (Bidirectional Encoder Representations from Transformers) era. In particular, recent works indicate that the contextual information from unstructured clinical notes benefits in particular longer-term sepsis prediction tasks [30]. It is worth stressing that the models mentioned above date back to the pre-Large-Language-Model (LLM) era. LLMs, such as GPT-4 and Med-PaLM, have emerged as powerful tools in healthcare, capable of processing and synthesizing complex clinical data. In the context of sepsis care, LLMs have the potential to enhance clinical decision support by rapidly analyzing unstructured data from electronic health records (EHRs), such as clinical notes, laboratory reports, and imaging findings, to assist in early detection and risk stratification [105]. By providing real-time summaries of patient status and highlighting critical findings, LLMs can support clinicians in timely decision-making, potentially improving early recognition and management of sepsis. Additionally, integration of LLMs into clinical workflows may streamline documentation and reduce cognitive load, allowing healthcare professionals to focus more on patient care [106]. These bright prospects should be contrasted with severe shortcomings of a direct application of LLMs as highlighted in a recent study in a critical care context [107] namely in part significantly worse performance levels compared to physicians, failure to following guidelines or to interpret certain kinds of clinical information such as lab values.

#### Omics data

**Fig. 1:**
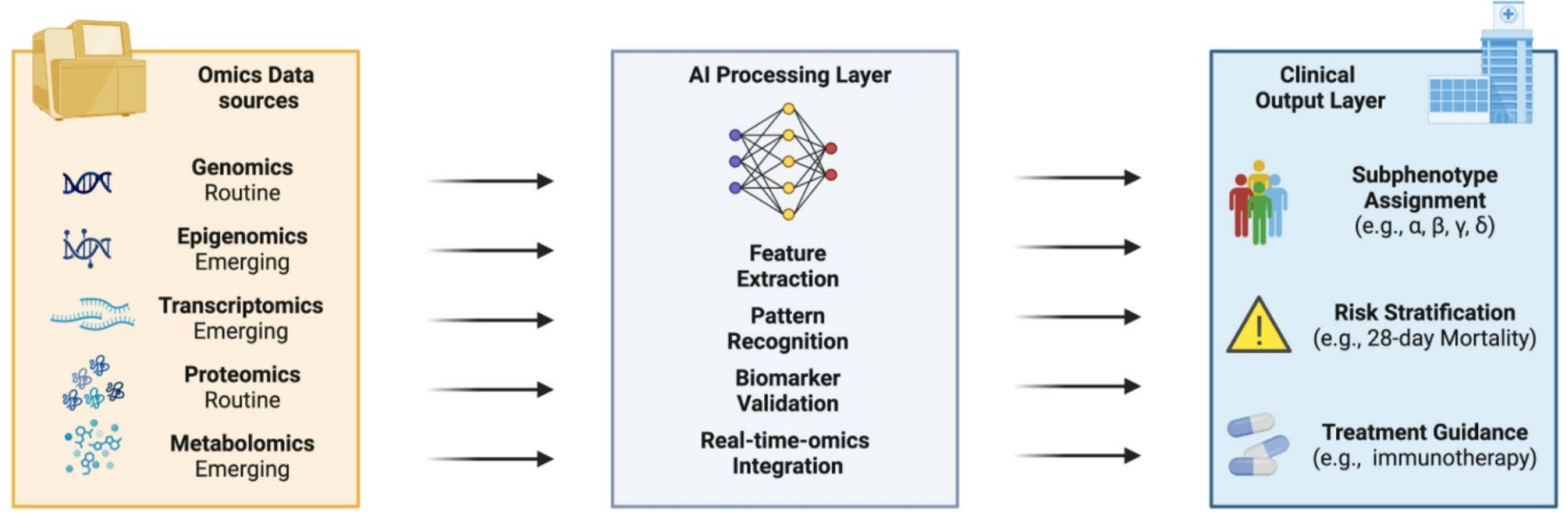
Conceptual framework for AI-driven integration of omics data in sepsis care. This schematic illustrates the layered workflow for precision medicine in sepsis, enabled by artificial intelligence (AI). Diverse omics data sources—including routine (genomics, proteomics) and emerging (epigenomics, transcriptomics, metabolomics) modalities—serve as inputs to an AI processing layer. This layer performs tasks such as feature extraction, pattern recognition, biomarker validation, and real-time integration of omics data. The resulting outputs support clinically relevant applications, including subphenotype assignment (e.g., α, β, γ, δ), risk stratification (e.g., 28-day mortality), and personalized treatment guidance (e.g., immunotherapy). This framework highlights the potential of AI to bridge molecular insights and clinical decision-making in sepsis management

The integration of omics diagnostics represents another approach, which could enhance sepsis prediction when combined with ML methods [108]. Omics technologies, such as genomics, proteomics, metabolomics, and transcriptomics, can revolutionize sepsis management by offering, more profound insights into the molecular basis of the disease and potentially revealing novel therapeutic targets. Through the analysis of patients’ omics data, clinicians can identify specific biomarkers associated with different sepsis phenotypes, guiding more targeted interventions. Figure 1 highlights the role of AI models in advancing biomarker validation and supporting personalized sepsis care through the integration of omics data. For example, genomics can reveal genetic predispositions to sepsis, while proteomics and metabolomics can identify biomarkers indicative of specific organ dysfunctions [109]. These advancements enable a more stratified treatment approach, with therapeutic decisions informed by each patient’s unique molecular profile [110, 111]. So far, the majority of omics diagnostics represent non-routine measurements and have rarely undergone prospective validation. With the emergence of omics markers, one can expect an expansion of this field, potentially leading to further breakthroughs in sepsis diagnosis and treatment.

As AI models continue to evolve, the integration of omics data and enhanced real-time reclassification capabilities offer the potential to deliver precision medicine strategies tailored to the dynamic and heterogeneous nature of sepsis. Real-time data integration will be vital in enhancing the precision of care. As patients’ conditions change, these models can dynamically update phenotypic classifications, ensuring that treatments remain responsive and aligned with the current clinical picture [112]. Such adaptability will allow clinicians to make more effective, timely decisions. The ability to detect complex patterns in large datasets makes advanced systems particularly valuable for personalized treatment. Incorporating molecular data will enable even more precise classifications, further advancing personalized medicine [20]. Moreover, dynamic reclassification of patients based on continuously updated data ensures that treatment strategies remain relevant as patients’ conditions evolve [113]. This minimizes the risks of overtreatment or undertreatment.

While AI models show promise in integrating multi-modal data, including omics, into sepsis management, the real-time clinical application of omics data remains limited. Current omics assays, such as genomics, proteomics, and metabolomics, typically require complex laboratory workflows and extended processing times, making them impractical for point-of-care use in acute settings. There is a lack of validated point-of-care testing (POCT) platforms capable of delivering real-time omics-based diagnostics in sepsis. However, advances in rapid diagnostic technologies, such as biosensors and POCT devices, have demonstrated potential for near real-time detection of sepsis-relevant biomarkers. For example, novel nanotechnology-based biosensors have achieved rapid detection of pathogens in serum samples within seconds [114], and POCT systems for biomarkers like C-reactive protein, procalcitonin, and interleukin-6 are increasingly used in neonatal sepsis management [115]. Additionally, the integration of POCT for basic biomarkers, such as lactate and full blood count parameters, has been successfully implemented in remote clinical settings to improve timely sepsis detection and triage [116]. While full multi-omics integration into real-time clinical workflows remains an area for future development, these advancements in rapid biomarker testing are important steps toward more responsive sepsis diagnostics.

#### Raw waveform data

Another category of promising input modalities comprises waveform data, which encompasses ECG, photoplethysmogram (PPG), and arterial blood pressure captured at high sampling frequencies. Waveform analysis approaches can be broadly categorized into feature-based and raw-time-series-based approaches. As of feature-based approaches, a limited set of expert features extracted from the waveform data, such as HRV in the case of ECG, are known to be very predictive and widely considered in predictive models. Recent work further demonstrates that brief segments of waveform data, often just five minutes, can yield interpretable features such as heart rate variability (HRV), pulse arrival time (PAT), and mean arterial pressure variability (MAPV), which are predictive of septic shock onset well before clinical recognition [117]. HRV features commonly include time-domain indices (e.g., SDNN, RMSSD), frequency-domain metrics (e.g., LF/HF ratio), and nonlinear measures (e.g., entropy, Poincaré plots), all of which reflect autonomic regulation and perfusion dynamics [118]. To extract such features and prepare data for AI models, preprocessing methods such as noise filtering, artifact removal, and peak detection are required. More advanced methods, such as tensor decomposition (e.g., Canonical Polyadic Decomposition), have been successfully applied to retain spatiotemporal structure in multivariate waveform streams, improving predictive performance while reducing data dimensionality [119].

In contrast to handcrafted features, raw waveforms capture a wide range of conditions that do not necessarily pertain to a single organ system. In AI-enhanced ECG studies, for example, deep learning models have demonstrated the ability to infer numerous cardiac and non-cardiac conditions, such as diabetes and cirrhosis, from a single ECG, even within a single unified model [120]. Notably, initial exploratory studies on sepsis prediction suggest that using the complete waveform data offers a more comprehensive source of information [121], in line with a recent study highlighting the added value of raw waveforms for general diagnostic tasks and patient deterioration tasks in the ED [122] widely across all considered tasks. A wide variety of deep learning architectures including convolutional neural networks (CNNs) and hybrid CNN–LSTM models have been successfully applied to capture spatiotemporal patterns in waveform dynamics. More recent works explore the use transformer models [123] or structured state-space models [122] to extract waveform representations that are informative for prediction tasks in a critical care context. Therefore, it seems very likely that including raw waveform data will also enhance predictive accuracy for sepsis and enable more nuanced patient stratification.

### Multimodality

Multimodality, which refers to the integration of diverse data types such as clinical, imaging, genomic, and sensor data, is an emerging paradigm in clinical prediction models [86]. A central challenge remains the necessity to circumvent the need for large training datasets covering several or in the worst case all modalities across for all samples, which would be indispensable for training a monolithic multimodal prediction model from scratch. Two solution components have recently emerged to address this challenge, the use of modular approaches with modality-specific encoder models combined with modality-specific pretraining. The resulting output representations of the different encoder models can then be combined using relatively shallow prediction models, as demonstrated in ICU/ED contexts [124, 125]. Pretraining encoder models on EHR data enable to build for example multimodal models that combine EHR data with OMICS data [126] while requiring only a few hundred samples with paired data from both modalities. In the context of sepsis, integrating multimodal data streams, including clinical observations, laboratory values, molecular biomarkers, and patient-generated health data, may enable real-time patient stratification and dynamic phenotyping. By integrating data sources such as genomics, proteomics, and wearable devices, advanced systems can continuously assess a patient’s condition, providing personalized treatment strategies that evolve with the patient’s health status [20]. Leveraging AI-supported systems biology to analyze dynamic interactions among genes, proteins, and metabolites has the potential to facilitate continuous assessment and guide personalized interventions in sepsis care in real-time [127].

### Generalist Medical AI in sepsis care

At present, the capabilities of sepsis prediction models remain quite limited. Nonetheless, there are undoubtedly promising prospects on the horizon for a generalist medical artificial intelligence [128]. Such a model, drawing upon medical expertise, can be constructed upon a foundational model meticulously pre-trained on extensive and diverse datasets equipped with both multimodal input and output capabilities. It could possess the capacity to address specific inquiries pertaining to individual patients. Not only would it retrieve similar cases, but it would also furnish therapeutic recommendations grounded in current guidelines and research literature. For instance, such systems could consolidate tasks currently handled by separate algorithms, such as optimizing antibiotic selection, de-escalation, and resistance prediction, central pillars of effective antibiotic stewardship in sepsis [129]. Early prototypes for such integrated systems, including reinforcement learning models and real-time decision support tools, have already demonstrated feasibility in clinical or simulated settings. To realize this vision, AI might need to acquire counterfactual reasoning skills, enabling it to evaluate the potential efficacy of particular therapeutic interventions. Moreover, generalist AI could leverage continuous waveform and hemodynamic data to improve fluid management decisions by predicting fluid responsiveness in real time, a challenge that remains poorly addressed in current sepsis care [130].

## Conclusions

**Fig. 2:**
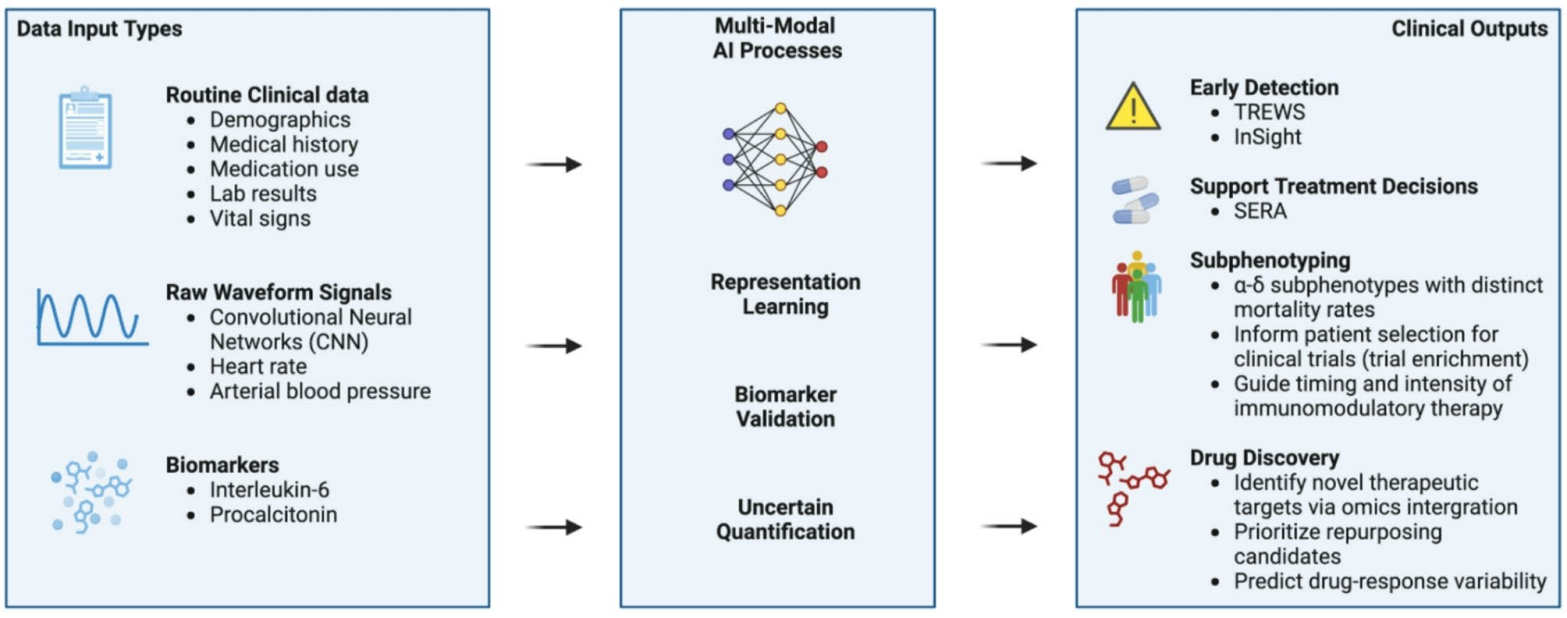
Multi-modal AI architecture for sepsis prediction, clinical support, and therapeutic innovation. This schematic illustrates how diverse input data—ranging from routine clinical variables and raw waveform signals to circulating biomarkers—can be integrated through advanced AI methods to support multiple downstream applications in sepsis care. The AI processing layer employs techniques such as representation learning, biomarker validation, and uncertainty quantification to derive clinically actionable insights. Outputs include early detection systems (e.g., TREWS, InSight), treatment decision support tools (e.g., SERA), and subphenotyping approaches that inform risk stratification, trial enrichment, and immunotherapy timing. Additionally, the framework highlights the emerging role of AI in drug discovery, including the identification of therapeutic targets, candidate repurposing, and personalized prediction of drug-response variability. This figure synthesizes the functional scope of AI across both bedside and translational domains in sepsis management

Artificial intelligence holds substantial promise for transforming sepsis management across three critical domains: early detection, patient phenotyping, and clinical decision support. While each of these applications addresses a distinct aspect of the clinical workflow, timeliness of intervention, understanding of biological heterogeneity, and optimization of therapeutic decisions, they are interdependent and must evolve in concert. Integrating insights across these domains is essential to fully leverage AI’s potential for improving sepsis outcomes (Fig. 2). To accelerate the safe and effective translation of AI into real-world practice, several priorities emerge. First, models must be rigorously validated in prospective clinical studies designed to reflect real-world complexity and meet regulatory expectations. Second, successful integration will require human-centered design strategies that involve clinicians and patients from the outset, ensuring usability, interpretability, and alignment with clinical workflows. Finally, adoption depends on economic feasibility and institutional readiness, issues that demand supportive reimbursement models and evidence of cost-effectiveness. Ultimately, the promise of AI in sepsis lies not in replacing clinical expertise, but in augmenting it with timely, data-driven insights. As the field matures, it will be critical to move beyond technical benchmarks and toward patient-centered outcomes, drawing on interdisciplinary collaboration and iterative learning. A deliberate focus on validation, usability, and equity will determine whether AI becomes a routine component of high-quality sepsis care.

## Data Availability

No datasets were generated or analysed during the current study.

## Abbreviations

AI: Artificial Intelligence
AMR: Antimicrobial Resistance
AUC: Area Under the Curve
COMPOSER: Context and Module–Based Prediction of Sepsis Risk
DTR: Dynamic Treatment Regimes
EHR: Electronic Health Record
eSOFA: Electronic Sequential Organ Failure Assessment
FDA: Food and Drug Administration
HIPAA: Health Insurance Portability and Accountability Act
ICU: Intensive Care Unit
LLM: Large Language Model
ML: Machine Learning
NLP: Natural Language Processing
OMICs: Omics Technologies (e.g., genomics, transcriptomics, proteomics, metabolomics)
POCT: Point–of–Care Testing
PPG: Photoplethysmogram
PPV: Positive Predictive Value
RCT: Randomized Controlled Trial
SOFA: Sequential Organ Failure Assessment
TREWS: Targeted Real–Time Early Warning System
XAI: Explainable Artificial Intelligence

## References

[1] Angus DC, van der Poll T. Severe sepsis and septic shock. N Engl J Med. 2013;369(9):840–51. 23984731 10.1056/NEJMra1208623

[2] Rudd KE, Johnson SC, Agesa KM, Shackelford KA, Tsoi DT, Kievlan DR, Colombara DV, Ikuta KS, Kisson N, Finfer S, et al. Global, regional, and National sepsis incidence and mortality, 1990–2017: analysis for the global burden of disease study. Lancet. 2020;395(10219):200–11. 31954465 10.1016/S0140-6736(19)32989-7 PMC6970225

[3] Stevenson EK, Rubenstein AR, Radin GT, Wiener RS, Walkey AJ. Two decades of mortality trends among patients with severe sepsis: a comparative meta-analysis. Crit Care Med. 2014;42(3):625–31. 24201173 10.1097/CCM.0000000000000026 PMC4313930

[4] Seymour CW, Gesten F, Prescott HC, Friedrich ME, Iwashyna TJ, Phillips GS, Lemeshow S, Osborn T, Terry KM, Levy MM. Time to treatment and mortality during mandated emergency care for sepsis. N Engl J Med. 2017;376(23):2235–44. 28528569 10.1056/NEJMoa1703058 PMC5538258

[5] Hotchkiss RS, Moldawer LL, Opal SM, Reinhart K, Turnbull IR, Vincent JL. Sepsis and septic shock. Nat Reviews Disease Primers. 2016;2:16045. 28117397 10.1038/nrdp.2016.45 PMC5538252

[6] Jentzer JC, Lawler PR, Van Houten HK, Yao X, Kashani KB, Dunlay SM. Cardiovascular events among survivors of sepsis hospitalization: A retrospective cohort analysis. J Am Heart Assoc. 2023;12(3):e027813. 36722388 10.1161/JAHA.122.027813 PMC9973620

[7] Nedeva C. Inflammation and cell death of the innate and adaptive immune system during sepsis. Biomolecules 2021, 11(7). 10.3390/biom11071011 10.3390/biom11071011 PMC8301842 34356636

[8] Iba T, Umemura Y, Wada H, Levy JH. Roles of coagulation abnormalities and microthrombosis in sepsis: pathophysiology, diagnosis, and treatment. Arch Med Res. 2021;52(8):788–97. 34344558 10.1016/j.arcmed.2021.07.003

[9] Cajander S, Kox M, Scicluna BP, Weigand MA, Mora RA, Flohe SB, Martin-Loeches I, Lachmann G, Girardis M, Garcia-Salido A, et al. Profiling the dysregulated immune response in sepsis: overcoming challenges to achieve the goal of precision medicine. Lancet Respir Med. 2024;12(4):305–22. 38142698 10.1016/S2213-2600(23)00330-2

[10] Im Y, Kang D, Ko RE, Lee YJ, Lim SY, Park S, Na SJ, Chung CR, Park MH, Oh DK, et al. Time-to-antibiotics and clinical outcomes in patients with sepsis and septic shock: a prospective nationwide multicenter cohort study. Crit Care. 2022;26(1):19. 35027073 10.1186/s13054-021-03883-0 PMC8756674

[11] Meyer NJ, Prescott HC. Sepsis and septic shock. N Engl J Med. 2024;391(22):2133–46. 39774315 10.1056/NEJMra2403213

[12] Collaborators GBDAR. Global burden of bacterial antimicrobial resistance 1990–2021: a systematic analysis with forecasts to 2050. Lancet. 2024;404(10459):1199–226. 39299261 10.1016/S0140-6736(24)01867-1 PMC11718157

[13] Vincent JL, van der Poll T, Marshall JC. The end of one size fits all sepsis therapies: toward an individualized approach. Biomedicines 2022, 10(9). 10.3390/biomedicines10092260 10.3390/biomedicines10092260 PMC9496597 36140361

[14] Evans L, Rhodes A, Alhazzani W, Antonelli M, Coopersmith CM, French C, Machado FR, McIntyre L, Ostermann M, Prescott HC, et al. Surviving sepsis campaign: international guidelines for management of sepsis and septic shock 2021. Crit Care Med. 2021;49(11):e1063–143. 34605781 10.1097/CCM.0000000000005337

[15] Ward PA. New approaches to the study of sepsis. EMBO Mol Med. 2012;4(12):1234–43. 23208733 10.1002/emmm.201201375 PMC3531600

[16] Im Y, Kang D, Ko RE, Lee YJ, Lim SY, Park S, Na SJ, Chung CR, Park MH, Oh DK et al. Time-to-antibiotics and clinical outcomes in patients with sepsis and septic shock: a prospective nationwide multicenter cohort study. Crit Care 2022, 26(1). 10.1186/s13054-021-03883-0 10.1186/s13054-021-03883-0 PMC8756674 35027073

[17] McMullan RR, McAuley DF, O’Kane CM, Silversides JA. Vascular leak in sepsis: physiological basis and potential therapeutic advances. Crit Care. 2024;28(1):97. 38521954 10.1186/s13054-024-04875-6 PMC10961003

[18] Williams B, Zou L, Pittet JF, Chao W. Sepsis-Induced coagulopathy: A comprehensive narrative review of pathophysiology, clinical presentation, diagnosis, and management strategies. Anesth Analg. 2024;138(4):696–711. 38324297 10.1213/ANE.0000000000006888 PMC10916756

[19] Gorecki GP, Tomescu DR, Ples L, Panaitescu AM, Dragosloveanu S, Scheau C, Sima RM, Coman IS, Grigorean VT, Cochior D. Implications of using artificial intelligence in the diagnosis of sepsis/sepsis shock. Germs. 2024;14(1):77–84. 39169980 10.18683/germs.2024.1419 PMC11333838

[20] Giamarellos-Bourboulis EJ, Aschenbrenner AC, Bauer M, Bock C, Calandra T, Gat-Viks I, Kyriazopoulou E, Lupse M, Monneret G, Pickkers P, et al. The pathophysiology of sepsis and precision-medicine-based immunotherapy. Nat Immunol. 2024;25(1):19–28. 38168953 10.1038/s41590-023-01660-5

[21] Li F, Wang S, Gao Z, Qing M, Pan S, Liu Y, Hu C. Harnessing artificial intelligence in sepsis care: advances in early detection, personalized treatment, and real-time monitoring. Front Med (Lausanne). 2024;11:1510792. 39835096 10.3389/fmed.2024.1510792 PMC11743359

[22] Stylianides C, Nicolaou A, Sulaiman WA, Alexandropoulou CA, Panagiotopoulos I, Karathanasopoulou K, Dimitrakopoulos G, Kleanthous S, Politi E, Ntalaperas D et al. AI advances in ICU with an emphasis on sepsis prediction: an overview. Mach Learn Know Extr 2025, 7(1). 10.3390/make7010006

[23] Agnello L, Vidali M, Padoan A, Lucis R, Mancini A, Guerranti R, Plebani M, Ciaccio M, Carobene A. Machine learning algorithms in sepsis. Clin Chim Acta. 2024;553:117738. 38158005 10.1016/j.cca.2023.117738

[24] Gao YL, Wang CL, Shen JX, Wang ZY, Liu YC, Chai YF. Systematic review and network meta-analysis of machine learning algorithms in sepsis prediction. Expert Syst Appl 2024, 245. 10.1016/j.eswa.2023.122982

[25] Wang D, Li J, Sun Y, Ding X, Zhang X, Liu S, Han B, Wang H, Duan X, Sun T. A machine learning model for accurate prediction of sepsis in ICU patients. Front Public Health. 2021;9:754348. 34722452 10.3389/fpubh.2021.754348 PMC8553999

[26] Calvert JS, Price DA, Chettipally UK, Barton CW, Feldman MD, Hoffman JL, Jay M, Das R. A computational approach to early sepsis detection. Comput Biol Med. 2016;74:69–73. 27208704 10.1016/j.compbiomed.2016.05.003

[27] Shimabukuro DW, Barton CW, Feldman MD, Mataraso SJ, Das R. Effect of a machine learning-based severe sepsis prediction algorithm on patient survival and hospital length of stay: a randomised clinical trial. BMJ Open Respir Res. 2017;4(1):e000234. 29435343 10.1136/bmjresp-2017-000234 PMC5687546

[28] Tekdos Seker Y, Cukurova Z, Ozel Bilgi D, Hergunsel O. Prognostic impact of early versus late initiation of renal replacement therapy based on early warning algorithm in critical care patients with acute kidney injury. Ther Apher Dial. 2020;24(4):445–52. 31661596 10.1111/1744-9987.13449

[29] Prasad V, Aydemir B, Kehoe IE, Kotturesh C, O’Connell A, Biebelberg B, Wang Y, Lynch JC, Pepino JA, Filbin MR, et al. Diagnostic suspicion bias and machine learning: breaking the awareness deadlock for sepsis detection. PLOS Digit Health. 2023;2(11):e0000365. 37910497 10.1371/journal.pdig.0000365 PMC10619833

[30] Goh KH, Wang L, Yeow AYK, Poh H, Li K, Yeow JJL, Tan GYH. Artificial intelligence in sepsis early prediction and diagnosis using unstructured data in healthcare. Nat Commun 2021, 12(1). 10.1038/s41467-021-20910-4 10.1038/s41467-021-20910-4 PMC7846756 33514699

[31] Schinkel M, van der Poll T, Wiersinga WJ. Artificial intelligence for early sepsis detection A word of caution. Am J Resp Crit Care. 2023;207(7):853–4. 10.1164/rccm.202212-2284VP PMC10111986 36724366

[32] Nemati S, Shashikumar SP, Holder AL, Wardi G, OR L. Randomized clinical trials or convenient controls: TREWS or FALSE? MedRxiv Preprint. 2022. 10.1038/s41591-41022-01894-41590.

[33] Goh KH, Wang L, Yeow AYK, Poh H, Li K, Yeow JJL, Tan GYH. Artificial intelligence in sepsis early prediction and diagnosis using unstructured data in healthcare. Nat Commun. 2021;12(1):711. 33514699 10.1038/s41467-021-20910-4 PMC7846756

[34] Adams R, Henry KE, Sridharan A, Soleimani H, Zhan A, Rawat N, Johnson L, Hager DN, Cosgrove SE, Markowski A, et al. Prospective, multi-site study of patient outcomes after implementation of the TREWS machine learning-based early warning system for sepsis. Nat Med. 2022;28(7):1455–60. 35864252 10.1038/s41591-022-01894-0

[35] Schinkel M, van der Poll T, Wiersinga WJ. Artificial intelligence for early sepsis detection: A word of caution. Am J Respir Crit Care Med. 2023;207(7):853–4. 36724366 10.1164/rccm.202212-2284VP PMC10111986

[36] Zhang Z, Chen L, Xu P, Wang Q, Zhang J, Chen K, Clements CM, Celi LA, Herasevich V, Hong Y. Effectiveness of automated alerting system compared to usual care for the management of sepsis. NPJ Digit Med. 2022;5(1):101. 35854120 10.1038/s41746-022-00650-5 PMC9296632

[37] van Calster B, Collins G, Vickers A, Wynants L, Kerr K, Barreñada L, Varoquaux G, Singh K, Moons K, Hernandez-Boussard T, et al. Performance evaluation of predictive AI models to support medical decisions: overview and guidance. ArXiv. 2024. 10.48550/ArXiv.42412.10288.36945687

[38] Komorowski M, Celi LA, Badawi O, Gordon AC, Faisal AA. The artificial intelligence clinician learns optimal treatment strategies for sepsis in intensive care. Nat Med. 2018;24(11):1716–20. 30349085 10.1038/s41591-018-0213-5

[39] Wong A, Otles E, Donnelly JP, Krumm A, McCullough J, DeTroyer-Cooley O, Pestrue J, Phillips M, Konye J, Penoza C, et al. External validation of a widely implemented proprietary sepsis prediction model in hospitalized patients. JAMA Intern Med. 2021;181(8):1065–70. 34152373 10.1001/jamainternmed.2021.2626 PMC8218233

[40] Boussina A, Shashikumar SP, Malhotra A, Owens RL, El-Kareh R, Longhurst CA, Quintero K, Donahue A, Chan TC, Nemati S, et al. Impact of a deep learning sepsis prediction model on quality of care and survival. NPJ Digit Med. 2024;7(1):14. 38263386 10.1038/s41746-023-00986-6 PMC10805720

[41] Yang J, Hao S, Huang J, Chen T, Liu R, Zhang P, Feng M, He Y, Xiao W, Hong Y, et al. The application of artificial intelligence in the management of sepsis. Med Rev (2021). 2023;3(5):369–80. 38283255 10.1515/mr-2023-0039 PMC10811352

[42] Seymour CW, Kennedy JN, Wang S, Chang CH, Elliott CF, Xu Z, Berry S, Clermont G, Cooper G, Gomez H, et al. Derivation, validation, and potential treatment implications of novel clinical phenotypes for sepsis. JAMA. 2019;321(20):2003–17. 31104070 10.1001/jama.2019.5791 PMC6537818

[43] Bhavani SV, Semler M, Qian ET, Verhoef PA, Robichaux C, Churpek MM, Coopersmith CM. Development and validation of novel sepsis subphenotypes using trajectories of vital signs. Intens Care Med. 2022;48(11):1582–92. 10.1007/s00134-022-06890-z PMC9510534 36152041

[44] Zhang Z, Chen L, Sun B, Ruan Z, Pan P, Zhang W, Jiang X, Zheng S, Cheng S, Xian L, et al. Identifying septic shock subgroups to tailor fluid strategies through multi-omics integration. Nat Commun. 2024;15(1):9028. 39424794 10.1038/s41467-024-53239-9 PMC11489719

[45] Komorowski M, Green A, Tatham KC, Seymour C, Antcliffe D. Sepsis biomarkers and diagnostic tools with a focus on machine learning. Ebiomedicine 2022, 86. 10.1016/j.ebiom.2022.104394 10.1016/j.ebiom.2022.104394 PMC9783125 36470834

[46] Festor P, Jia Y, Gordon AC, Faisal AA, Habli I, Komorowski M. Assuring the safety of AI-based clinical decision support systems: a case study of the AI clinician for sepsis treatment. BMJ Health Care Inf 2022, 29(1). 10.1136/bmjhci-2022-100549 10.1136/bmjhci-2022-100549 PMC9289024 35851286

[47] Komorowski M, Celi L, Badawi O, Gordon AC, Faisal AA. The artificial intelligence clinician learns optimal treatment strategies for sepsis in intensive care. Nat Med. 2018;24(11):1716–. 30349085 10.1038/s41591-018-0213-5

[48] Kalimouttou A, Kennedy JN, Feng J, Singh H, Saria S, Angus DC, Seymour CW, Pirracchio R. Optimal vasopressin initiation in septic shock: the OVISS reinforcement learning study. JAMA. 2025;333(19):1688–98. 40098600 10.1001/jama.2025.3046 PMC11920879

[49] Gupta A, Liu T, Shepherd S. Clinical decision support system to assess the risk of sepsis using tree augmented bayesian networks and electronic medical record data. Health Inf J. 2020;26(2):841–61. 10.1177/1460458219852872 31195874

[50] Jeon E, Choi JH, Suk HI. ADT(2)R: adaptive decision transformer for dynamic treatment regimes in sepsis. IEEE Trans Neural Netw Learn Syst 2024, PP. 10.1109/TNNLS.2024.3442243 10.1109/TNNLS.2024.3442243 39208049

[51] Singer M, Deutschman CS, Seymour CW, Shankar-Hari M, Annane D, Bauer M, Bellomo R, Bernard GR, Chiche JD, Coopersmith CM, et al. The third international consensus definitions for sepsis and septic shock (Sepsis-3). JAMA. 2016;315(8):801–10. 26903338 10.1001/jama.2016.0287 PMC4968574

[52] Rhee C, Zhang ZL, Wang R, Kadri S, Fram D, Schaaf R, Klompas M. Sepsis surveillance using sofa versus Esofa criteria based on routine Ehr data. Crit Care Med. 2018;46(1):689–689.

[53] Fixler A, Oliaro B, Frieden M, Girardo C, Winterbottom FA, Fort LB, Hill J. Alert to action: implementing artificial Intelligence-Driven clinical decision support tools for sepsis. Ochsner J. 2023;23(3):222–31. 37711478 10.31486/toj.22.0098 PMC10498958

[54] van der Vegt AH, Scott IA, Dermawan K, Schnetler RJ, Kalke VR, Lane PJ. Deployment of machine learning algorithms to predict sepsis: systematic review and application of the SALIENT clinical AI implementation framework. J Am Med Inf Assoc. 2023;30(7):1349–61. 10.1093/jamia/ocad075 PMC10280361 37172264

[55] Schinkel M, Nanayakkara PWB, Wiersinga WJ. Sepsis performance improvement programs: from evidence toward clinical implementation. Crit Care. 2022;26(1):77. 35337358 10.1186/s13054-022-03917-1 PMC8951662

[56] Moor M, Bennett N, Plecko D, Horn M, Rieck B, Meinshausen N, Bühlmann P, Borgwardt K. Predicting sepsis using deep learning across international sites: a and validation. Eclinicalmedicine 2023, 62. 10.1016/j.eclinm.2023.102124 10.1016/j.eclinm.2023.102124 PMC10425671 37588623

[57] Boussina A, Shashikumar SP, Malhotra A, Owens RL, El-Kareh R, Longhurst CA, Quintero K, Donahue A, Chan TC, Nemati S et al. Impact of a deep learning sepsis prediction model on quality of care and survival. Npj Digit Med 2024, 7(1). 10.1038/s41746-023-00986-6 10.1038/s41746-023-00986-6 PMC10805720 38263386

[58] Adams R, Henry KE, Sridharan A, Soleimani H, Zhan AD, Rawat N, Johnson L, Hager DN, Cosgrove SE, Markowski A, et al. Prospective, multi-site study of patient outcomes after implementation of the TREWS machine learning-based early warning system for sepsis. Nat Med. 2022;28(7):1455–. 35864252 10.1038/s41591-022-01894-0

[59] Kheterpal S, Singh K, Topol EJ. Digital medicine digitising the prediction and management of sepsis. Lancet. 2022;399(10334):1459–1459. 35430013 10.1016/S0140-6736(22)00658-4

[60] Wong A, Otles E, Donnelly JP. External validation of a widely implemented proprietary sepsis prediction model in hospitalized patients (181, Pg 1065, 2021). Jama Intern Med. 2021;181(8):1144–1144. 10.1001/jamainternmed.2021.2626 PMC8218233 34152373

[61] Joshi G, Jain A, Araveeti SR, Adhikari S, Garg H, Bhandari M. FDA-Approved artificial intelligence and machine learning (AI/ML)-Enabled medical devices: an updated landscape. Electronics-Switz 2024, 13(3). 10.3390/electronics13030498

[62] Doutreligne M, Struja T, Abecassis J, Morgand C, Celi LA, Varoquaux G. Step-by-step causal analysis of EHRs to ground decision-making. PLOS Digit Health. 2025;4(2):e0000721. 39899627 10.1371/journal.pdig.0000721 PMC11790099

[63] Van Vogt E, Gordon AC, Diaz-Ordaz K, Cro S. Application of causal forests to randomised controlled trial data to identify heterogeneous treatment effects: a case study. BMC Med Res Methodol. 2025;25(1):50. 39987431 10.1186/s12874-025-02489-2 PMC11846376

[64] Lam TYT, Cheung MFK, Munro YL, Lim KM, Shung D, Sung JJY. Randomized controlled trials of artificial intelligence in clinical practice: systematic review. J Med Internet Res. 2022;24(8):e37188. 35904087 10.2196/37188 PMC9459941

[65] Bhargava A, López-Espina C, Schmalz L, Khan S, Watson GL, Urdiales D, Updike L, Kurtzman N, Dagan A, Doodlesack A et al. FDA-Authorized AI/ML tool for sepsis prediction: development and validation. NEJM AI 2024:10.1056/AIoa2400867

[66] Muralidharan V, Adewale BA, Huang CJ, Nta MT, Ademiju PO, Pathmarajah P, Hang MK, Adesanya O, Abdullateef RO, Babatunde AO, et al. A scoping review of reporting gaps in FDA-approved AI medical devices. NPJ Digit Med. 2024;7(1):273. 39362934 10.1038/s41746-024-01270-x PMC11450195

[67] Persson I, Macura A, Becedas D, Sjovall F. Early prediction of sepsis in intensive care patients using the machine learning algorithm NAVOY(R) sepsis, a prospective randomized clinical validation study. J Crit Care. 2024;80:154400. 38245375 10.1016/j.jcrc.2023.154400

[68] Shashikumar SP, Mohammadi S, Krishnamoorthy R, Patel A, Wardi G, Ahn JC, Singh K, Aronoff-Spencer E, Nemati S. Development and prospective implementation of a large Language model based system for early sepsis prediction. NPJ Digit Med. 2025;8(1):290. 40379845 10.1038/s41746-025-01689-w PMC12084535

[69] Valan B, Prakash A, Ratliff W, Gao M, Muthya S, Thomas A, Eaton JL, Gardner M, Nichols M, Revoir M, et al. Evaluating sepsis watch generalizability through multisite external validation of a sepsis machine learning model. NPJ Digit Med. 2025;8(1):350. 40500319 10.1038/s41746-025-01664-5 PMC12159134

[70] Gichoya JW, Thomas K, Celi LA, Safdar N, Banerjee I, Banja JD, Seyyed-Kalantari L, Trivedi H, Purkayastha S. AI pitfalls and what not to do: mitigating bias in AI. Br J Radiol. 2023;96(1150):20230023. 37698583 10.1259/bjr.20230023 PMC10546443

[71] Yesil MR, Talli I, Pelloso M, Cosma C, Pangrazzi E, Plebani M, Ustundag Y, Padoan A. Impact of analytical bias on machine learning models for sepsis prediction using laboratory data. Clin Chem Lab Med 2025. 10.1515/cclm-2025-0491 10.1515/cclm-2025-0491 40440484

[72] Wagner P, Mehari T, Haverkamp W, Strodthoff N. Explaining deep learning for ECG analysis: Building blocks for auditing and knowledge discovery. Comput Biol Med. 2024;176:108525. 38749322 10.1016/j.compbiomed.2024.108525

[73] Ghassemi M, Oakden-Rayner L, Beam AL. The false hope of current approaches to explainable artificial intelligence in health care. Lancet Digit Health. 2021;3(11):e745–50. 34711379 10.1016/S2589-7500(21)00208-9

[74] Goncalves LS, Amaro MLM, Romero ALM, Schamne FK, Fressatto JL, Bezerra CW. Implementation of an artificial intelligence algorithm for sepsis detection. Rev Bras Enferm. 2020;73(3):e20180421. 32294705 10.1590/0034-7167-2018-0421

[75] Hassan N, Slight R, Weiand D, Vellinga A, Morgan G, Aboushareb F, Slight SP. Preventing sepsis; how can artificial intelligence inform the clinical decision-making process? A systematic review. Int J Med Inf. 2021;150:104457. 10.1016/j.ijmedinf.2021.104457 33878596

[76] Aslan AT, Permana B, Harris PNA, Naidoo KD, Pienaar MA, Irwin AD. The opportunities and challenges for artificial intelligence to improve sepsis outcomes in the paediatric intensive care unit. Curr Infect Dis Rep. 2023;25(11):243–53.

[77] Dusing C, Cimiano P, Rehberg S, Scherer C, Kaup O, Koster C, Hellmich S, Herrmann D, Meier KL, Classen S, et al. Integrating federated learning for improved counterfactual explanations in clinical decision support systems for sepsis therapy. Artif Intell Med. 2024;157:102982. 39277983 10.1016/j.artmed.2024.102982

[78] Sullivan BA, Beam K, Vesoulis ZA, Aziz KB, Husain AN, Knake LA, Moreira AG, Hooven TA, Weiss EM, Carr NR, et al. Transforming neonatal care with artificial intelligence: challenges, ethical consideration, and opportunities. J Perinatol. 2024;44(1):1–11. 38097685 10.1038/s41372-023-01848-5 PMC10872325

[79] Banja JD, Xie Y, Smith JR, Rana S, Holder AL: Mitigating bias in machine learning models with Ethics-Based initiatives: the case of sepsis. Am J Bioeth 2025:1–14. 10.1080/15265161.2025.2497971 10.1080/15265161.2025.2497971 PMC12353398 40354171

[80] Struja T, Matos J, Lam B, Cao Y, Liu X, Chan Z, Jia Y, Sauer CM, D’Couto H, Dankwa-Mullan I, et al. Evaluating equitable care in the icu:creating a causal inference template to assess the impact of life-sustaining interventions across Racial and ethnic groups. Heart Lung. 2025;72:48–56. 40163946 10.1016/j.hrtlng.2025.03.011

[81] Lam JY, Boussina A, Shashikumar SP, Owens RL, Nemati S, Josef CS. The impact of laboratory data missingness on sepsis diagnosis timeliness. Jamia Open 2024, 7(3). 10.1093/jamiaopen/ooae085 10.1093/jamiaopen/ooae085 PMC11418648 39314673

[82] Iqbal F, Chandra P, Khan AA, Lewis LES, Acharya D, Vandana KE, Jayashree P, Shenoy PA. Prediction of mortality among neonates with sepsis in the neonatal intensive care unit: A machine learning approach. Clin Epidemiol Glob 2023, 24. 10.1016/j.cegh.2023.101414

[83] Bomrah S, Uddin M, Upadhyay U, Komorowski M, Priya J, Dhar E, Hsu SC, Syed-Abdul S. A scoping review of machine learning for sepsis prediction- feature engineering strategies and model performance: a step towards explainability. Crit Care 2024, 28(1). 10.1186/s13054-024-04948-6 10.1186/s13054-024-04948-6 PMC11131234 38802973

[84] Eisenberg MA, Balamuth F. Pediatric sepsis screening in US hospitals. Pediatr Res. 2022;91(2):351–8. 34417563 10.1038/s41390-021-01708-y PMC8378117

[85] Lamproudis A, Henriksson A, Valik JK, Naucler P. Improving the timeliness of early prediction models for sepsis through utility optimization. Proc Int C Tools Art 2022:1062–9. 10.1109/ICTAI56018.2022.00162

[86] Acosta JN, Falcone GJ, Rajpurkar P, Topol EJ. Multimodal biomedical AI. Nat Med. 2022;28(9):1773–84. 36109635 10.1038/s41591-022-01981-2

[87] van den Berg M, van Beuningen FE, Ter Maaten JC, Bouma HR. Hospital-related costs of sepsis around the world: A systematic review exploring the economic burden of sepsis. J Crit Care. 2022;71:154096. 35839604 10.1016/j.jcrc.2022.154096

[88] Ramesh K, Boussina A, Shashikumar SP, Malhotra A, Longhurst CA, Josef CS, Quintero K, Del Rosso J, Nemati S, Wardi G. Quantifying healthcare provider perceptions of a novel deep learning algorithm to predict sepsis: electronic survey. Crit Care Explor. 2025;7(6):e1276. 40466050 10.1097/CCE.0000000000001276 PMC12140755

[89] Zhang S, Yu J, Xu X, Yin C, Lu Y, Yao B, Tory M, Padilla LM, Caterino J, Zhang P et al. Rethinking Human-AI Collaboration in Complex Medical Decision Making: A Case Study in Sepsis Diagnosis. *Proc SIGCHI Conf Hum Factor Comput Syst* 2024, 2024. 10.1145/3613904.3642343 PMC11149368 38835626

[90] Rogers P, Boussina AE, Shashikumar SP, Wardi G, Longhurst CA, Nemati S. Optimizing the implementation of clinical predictive models to minimize National costs: sepsis case study. J Med Internet Res. 2023;25:e43486. 36780203 10.2196/43486 PMC9972209

[91] Zwerwer LR, van der Pol S, Zacharowski K, Postma MJ, Kloka J, Friedrichson B, van Asselt ADI. The value of artificial intelligence for the treatment of mechanically ventilated intensive care unit patients: an early health technology assessment. J Crit Care. 2024;82:154802. 38583302 10.1016/j.jcrc.2024.154802

[92] Saeed U, Insaf RA, Piracha ZZ, Tariq MN, Sohail A, Abbasi UA, Fida Rana MS, Gilani SS, Noor S, Noor E, et al. Crisis averted: a world united against the menace of multiple drug-resistant superbugs -pioneering anti-AMR vaccines, RNA interference, nanomedicine, CRISPR-based antimicrobials, bacteriophage therapies, and clinical artificial intelligence strategies to safeguard global antimicrobial arsenal. Front Microbiol. 2023;14:1270018. 38098671 10.3389/fmicb.2023.1270018 PMC10720626

[93] Denecke K, Baudoin CR. A review of artificial intelligence and robotics in transformed health ecosystems. Front Med-Lausanne 2022, 9. 10.3389/fmed.2022.795957 10.3389/fmed.2022.795957 PMC9299071 35872767

[94] Choi YB, Capitan KE, Krause JS, Streeper MM. Challenges associated with privacy in health care industry: implementation of HIPAA and the security rules. J Med Syst. 2006;30(1):57–64. 16548416 10.1007/s10916-006-7405-0

[95] Peregrin T. Managing HIPAA compliance includes legal and ethical considerations. J Acad Nutr Diet. 2021;121(2):327–9. 33487218 10.1016/j.jand.2020.11.012

[96] Pan W, Xu Z, Rajendran S, Wang F. An adaptive federated learning framework for clinical risk prediction with electronic health records from multiple hospitals. Patterns (N Y). 2024;5(1):100898. 38264713 10.1016/j.patter.2023.100898 PMC10801228

[97] Mandl KD, Perakslis ED. HIPAA and the leak of deidentified EHR data. N Engl J Med. 2021;384(23):2171–3. 34110112 10.1056/NEJMp2102616

[98] Petersson L, Vincent K, Svedberg P, Nygren JM, Larsson I. Ethical considerations in implementing AI for mortality prediction in the emergency department: linking theory and practice. Digit Health 2023, 9. 10.1177/20552076231206588 10.1177/20552076231206588 PMC10566278 37829612

[99] Santacroce E, D’Angerio M, Ciobanu AL, Masini L, Lo Tartaro D, Coloretti I, Busani S, Rubio I, Meschiari M, Franceschini E et al.: advances and challenges in sepsis management: modern tools and future directions. Cells 2024, 13(5). 10.3390/cells13050439 10.3390/cells13050439 PMC10931424 38474403

[100] Boussina A, Wardi G, Shashikumar SP, Malhotra A, Zheng K, Nemati S. Representation learning and spectral clustering for the development and external validation of dynamic sepsis phenotypes: observational cohort study. J Med Internet Res 2023, 25. 10.2196/45614 10.2196/45614 PMC10337434 37351927

[101] Fleuren LM, Klausch TLT, Zwager CL, Schoonmade LJ, Guo TJ, Roggeveen LF, Swart EL, Girbes ARJ, Thoral P, Ercole A, et al. Machine learning for the prediction of sepsis: a systematic review and meta-analysis of diagnostic test accuracy. Intens Care Med. 2020;46(3):383–400. 10.1007/s00134-019-05872-y PMC7067741 31965266

[102] Yadgarov MY, Landoni G, Berikashvili LB, Polyakov PA, Kadantseva KK, Smirnova AV, Kuznetsov IV, Shemetova MM, Yakovlev AA, Likhvantsev VV. Early detection of sepsis using machine learning algorithms: a systematic review and network meta-analysis. Front Med-Lausanne 2024, 11. 10.3389/fmed.2024.1491358 10.3389/fmed.2024.1491358 PMC11523135 39478824

[103] Chen E, Kansal A, Chen J, Jin BT, Reisler JR, Kim DA, Rajpurkar P. Multimodal clinical benchmark for emergency care (MC-BEC): A comprehensive benchmark for evaluating foundation models in emergency medicine. Adv Neur *In* 2023. 10.48550/arXiv.2311.04937

[104] Yan MY, Gustad LT, Nytro O. Sepsis prediction, early detection, and identification using clinical text for machine learning: a systematic review. J Am Med Inf Assn. 2022;29(3):559–75. 10.1093/jamia/ocab236 PMC8800516 34897469

[105] Li Q, Ma HB, Song D, Bai YP, Zhao LN, Xie KL. Early prediction of sepsis using chatGPT-generated summaries and structured data. Multimed Tools Appl 2024. 10.1007/s11042-024-18378-7

[106] Aityan SK, Mosaddegh A, Herrero R, Inchingolo F, Nguyen KCD, Balzanelli M, Lazzaro R, Iacovazzo N, Cefalo A, Carriero L et al. Integrated AI Medical Emergency Diagnostics Advising System. *Electronics-Switz* 2024, 13(22).

[107] Hager P, Jungmann F, Holland R, Bhagat K, Hubrecht I, Knauer M, Vielhauer J, Makowski M, Braren R, Kaissis G et al. Evaluation and mitigation of the limitations of large Language models in clinical decision-making. Nat Med 2024, 30(9). 10.1038/s41591-024-03097-1 10.1038/s41591-024-03097-1 PMC11405275 38965432

[108] Komorowski M, Green A, Tatham KC, Seymour C, Antcliffe D. Sepsis biomarkers and diagnostic tools with a focus on machine learning. EBioMedicine. 2022;86:104394. 36470834 10.1016/j.ebiom.2022.104394 PMC9783125

[109] Huang PF, Liu YQ, Li Y, Xin Y, Nan CC, Luo YH, Feng YT, Jin NN, Peng YH, Wang DW et al. Metabolomics- and proteomics-based multi-omics integration reveals early metabolite alterations in sepsis-associated acute kidney injury. Bmc Med 2025, 23(1). 10.1186/s12916-025-03920-7 10.1186/s12916-025-03920-7 PMC11818193 39934788

[110] Mi YX, Burnham KL, Charles PD, Heilig R, Vendrell I, Whalley J, Torrance HD, Antcliffe DB, May SM, Neville MJ et al. High-throughput mass spectrometry maps the sepsis plasma proteome and differences in patient response. Sci Transl Med 2024, 16(750). 10.1126/scitranslmed.adh0185 10.1126/scitranslmed.adh0185 38838133

[111] Shankar-Hari M, Calandra T, Soares MP, Bauer M, Wiersinga WJ, Prescott HC, Knight JC, Baillie KJ, Bos LDJ, Derde LPG, et al. Reframing sepsis immunobiology for translation: towards informative subtyping andtargeted Immunomodulatory therapies. Lancet Resp Med. 2024;12(4):323–36. 10.1016/S2213-2600(23)00468-X PMC11025021 38408467

[112] Boussina A, Wardi G, Shashikumar SP, Malhotra A, Zheng K, Nemati S. Representation learning and spectral clustering for the development and external validation of dynamic sepsis phenotypes: observational cohort study. J Med Internet Res. 2023;25:e45614. 37351927 10.2196/45614 PMC10337434

[113] Sendak MP, Ratliff W, Sarro D, Alderton E, Futoma J, Gao M, Nichols M, Revoir M, Yashar F, Miller C, et al. Real-World integration of a sepsis deep learning technology into routine clinical care: implementation study. JMIR Med Inf. 2020;8(7):e15182. 10.2196/15182 PMC7391165 32673244

[114] Sheini A. A point-of-care testing sensor based on fluorescent nanoclusters for rapid detection of septicemia in children. Sens Actuat B-Chem 2021, 328. 10.1016/j.snb.2020.129029

[115] Goyal M, Mascarenhas D, Prashanth RR, Haribalakrishna A. Diagnostic accuracy of Point-of-Care testing of C-Reactive protein, Interleukin-6, and procalcitonin in neonates with clinically suspected sepsis: A prospective observational study. Med Prin Pract. 2024;33(3):291–8. 10.1159/000536678 PMC11175603 38320541

[116] Spaeth B, Taylor S, Shephard M, Reed RL, Omond R, Karnon J, Bonevski B, Rissel C, Ullah S, Noutsos T, et al. Point-of-care testing for sepsis in remote Australia and for first nations peoples. Nat Med. 2024;30(8):2105–6. 38816610 10.1038/s41591-024-03034-2

[117] Soliman MM, Marshall C, Kimball JP, Choudhary T, Clermont G, Pinsky MR, Buchman TG, Coopersmith CM, Inan OT, Kamaleswaran R. Parsimonious Waveform-derived features consisting of pulse arrival time and heart rate variability predicts the onset of septic shock. Biomed Signal Process Control 2024, 92. 10.1016/j.bspc.2024.105974 10.1016/j.bspc.2024.105974 PMC10977921 38559667

[118] Apalak M, Kiasaleh K. Advancing early detection of sepsis with Temporal convolutional networks using ECG signals. Ieee Access. 2024;12:3417–27.

[119] Alge OP, Pickard J, Zhang W, Cheng S, Derksen H, Omenn GS, Gryak J, VanEpps JS, Najarian K. Continuous sepsis trajectory prediction using tensor-reduced physiological signals. Sci Rep. 2024;14(1):18155. 39103488 10.1038/s41598-024-68901-x PMC11300462

[120] Strodthoff N, Alcaraz JML, Haverkamp W. Prospects for artificial intelligence-enhanced electrocardiogram as a unified screening tool for cardiac and non-cardiac conditions: an explorative study in emergency care. Eur Heart J-Digit Hl. 2024;5(4):454–60. 10.1093/ehjdh/ztae039 PMC11284007 39081937

[121] Mataraso SJ, Espinosa CA, Seong D, Reincke SM, Berson E, Reiss JD, Kim Y, Ghanem M, Shu CH, James T, et al. A machine learning approach to leveraging electronic health records for enhanced omics analysis. Nat Mach Intell. 2025;7(2):293–306. 40008295 10.1038/s42256-024-00974-9 PMC11847705

[122] Alcaraz JML, Bouma H, Strodthoff N. Enhancing clinical decision support with physiological waveforms - A multimodal benchmark in emergency care. Comput Biol Med. 2025;192(Pt A):110196. 40311469 10.1016/j.compbiomed.2025.110196

[123] Sundrani S, Chen J, Jin BT, Abad ZSH, Rajpurkar P, Kim D. Predicting patient decompensation from continuous physiologic monitoring in the emergency department. NPJ Digit Med. 2023;6(1):60. 37016152 10.1038/s41746-023-00803-0 PMC10073111

[124] Soenksen LR, Ma Y, Zeng C, Boussioux L, Carballo KV, Na LY, Wiberg HM, Li ML, Fuentes I, Bertsimas D. Integrated multimodal artificial intelligence framework for healthcare applications. Npj Digit Med 2022, 5(1). 10.1038/s41746-022-00689-4 10.1038/s41746-022-00689-4 PMC9489871 36127417

[125] Sundrani S, Chen JL, Jin BT, Abad ZSH, Rajpurkar P, Kim D. Predicting patient decompensation from continuous physiologic monitoring in the emergency department. Npj Digit Med 2023, 6(1). 10.1038/s41746-023-00803-0 10.1038/s41746-023-00803-0 PMC10073111 37016152

[126] Mataraso SJ, Espinosa CA, Seong D, Reincke SM, Berson E, Reiss JD, Kim Y, Ghanem M, Shu CH, James T et al. A machine learning approach to leveraging electronic health records for enhanced omics analysis. Nat Mach Intell 2025, 7(2). 10.1038/s42256-024-00974-9 10.1038/s42256-024-00974-9 PMC11847705 40008295

[127] Zhao TY, Liu JX, Zeng X, Wang W, Li S, Zang TY, Peng JJ, Yang Y. Prediction and collection of protein-metabolite interactions. Brief Bioinform 2021, 22(5). 10.1093/bib/bbab014 10.1093/bib/bbab014 33554247

[128] Moor M, Banerjee O, Abad ZSH, Krumholz HM, Leskovec J, Topol EJ, Rajpurkar P. Foundation models for generalist medical artificial intelligence. Nature. 2023;616(7956):259–65. 37045921 10.1038/s41586-023-05881-4

[129] AlGain S, Marra AR, Kobayashi T, Marra PS, Celeghini PD, Hsieh MK, Shatari MA, Althagafi S, Alayed M, Ranavaya JI, et al. Can we rely on artificial intelligence to guide antimicrobial therapy? A systematic literature review. Antimicrob Steward Healthc Epidemiol. 2025;5(1):e90. 40226293 10.1017/ash.2025.47 PMC11986881

[130] Gupta CB, Basu D, Williams TK, Neff LP, Johnson MA, Patel NT, Ganapathy AS, Lane MR, Radaei F, Chuah CN, et al. Improving the precision of shock resuscitation by predicting fluid responsiveness with machine learning and arterial blood pressure waveform data. Sci Rep. 2024;14(1):2227. 38278825 10.1038/s41598-023-50120-5 PMC10817926

